# Protective effects of betaine on the early fatty liver in laying hens through ameliorating lipid metabolism and oxidative stress

**DOI:** 10.3389/fnut.2024.1505357

**Published:** 2024-11-25

**Authors:** Chaohui Wang, Xi Sun, Xiaoying Liu, Yumeng Wang, Jiarui Luo, Xiaojun Yang, Yanli Liu

**Affiliations:** College of Animal Science and Technology, Northwest A&F University, Yangling, China

**Keywords:** betaine, fatty liver, laying hens, metabolomics, transcriptomics

## Abstract

**Introduction:**

Fatty liver syndrome (FLS) is a prevalent nutritional and metabolic disease that mainly occurs in caged laying hens, causing substantial losses in the poultry industry. The study was carried out to explore the protective effect and potential mechanism of betaine on early FLS.

**Methods:**

There were three groups: Con group (basal diet), FLS group (Dexamethasone injection + basal diet) and betaine group (Dexamethasone injection + basal diet with 8 g/kg betaine). Birds in FLS and betaine groups were treated with subcutaneous dexamethasone injection once a day at a dosage of 4.50 mg/kg body weight for 7 days.

**Results:**

The results revealed that DXM treatment significantly increased the liver index, serum aspartate aminotransferase (AST), total protein (TP), total bilirubin (TBIL), total biliary acid (TBA), total cholesterol (TC), high density lipoprotein cholesterol (HDL-c), low density lipoprotein cholesterol (LDL-c), and glucose (GLU) (*p* < 0.05). Additionally, hepatic TC and TG levels were also elevated (*p* < 0.05). Meanwhile, H&E and oil red O staining showed that there were a large number of vacuoles and lipid droplets in the liver of hens in FLS group. Dietary betaine addition significantly alleviated the increasing of serum TBIL, TBA and hepatic TC caused by dexamethasone treatment (*p* < 0.05). There existed 1,083 up- and 996 down-regulated genes in FLS group when compared with the control, and there were 169 upregulation and 405 downregulation genes in BT group when compared with FLS group. A total of 37 differential expression genes (DEGs) were rescued by betaine addition, which were related to lipid metabolism and antioxidant functions including APOC3, APOA4, G0S2, ERG28, PLA2G3, GPX4 and SLC5A8. Serum metabolomics analysis showed that 151 differential metabolites were identified in FLS group when compared with the control. Dietary betaine addition could rescue the changes of metabolites partly such as chicoric acid, gamma-aminobutyric acid, linoleic acid, telmisartan, which were associated with anti-oxidative function. In addition, RT-PCR results showed that genes involved in lipid metabolism, such as ACC, FAS, SCD1, ELOVL6, SREBP1, GR, ATGL and MTTP were markedly upregulated at the mRNA level (*p* < 0.05). However, dietary supplementation with betaine can reversed the expression of these genes (*p* < 0.05). Importantly, dietary betaine supplementation could reverse increased lipid synthesis partly by regulating PI3K/AKT/SREBP and CEBPα pathways in the liver based on western blot results (*p* < 0.05).

**Conclusion:**

Dexamethasone treatment could establish the early FLS model in laying hens with hepatic lipid accumulation and no inflammation, which could be attenuated by dietary betaine addition.

## 1 Introduction

Fatty liver syndrome (FLS) is a common nutritional metabolic disease in laying hens during intensive cage rearing. It is characterized by excessive fat accumulation in the liver, which adversely affects overall production performance and egg production rate (1, 2). Studies have reported that FLS can contribute significantly to the mortality rate in laying hens accounting for up to 74% especially in caged condition (3, 4). All of these bring substantial economic losses for laying hen industry. Therefore, investigating the pathogenesis of FLS and identifying effective preventive measures are crucial to reduce the incidence of FLS and prevent it.

To date, most FLS models have been induced through long-term dietary interventions (5–7). Additionally, some researchers have employed gene editing methods to establish FLS models (8–10), which require substantial investment. Dexamethasone (DXM), a synthetic glucocorticoid, is commonly employed as an anti-inflammatory and immunosuppressive medication (11). It was reported that DXM treatment can induce lipid metabolism disorders within a relatively short time (12, 13). Furthermore, a recent study emphasized the importance of focusing on the early stage of FLS, as it could be reversibly regulated during the stage of simple steatosis (14). Consequently, DXM was selected for early FLS model construction due to its anti-inflammatory properties and the relatively short duration of treatment in the current study.

Betaine assumes a pivotal role in the folate/methionine cycle as a methyl donor and is involved in regulating the gene expression through DNA methylation and histone modification (15, 16). A growing number of studies have demonstrated that betaine can notably enhance the production performance of broilers (17) and increase the laying rate of laying hens (18, 19). Additionally, betaine has been shown to reduce serum triglyceride (TG) levels in laying hens (20). Another study has shown that the addition of betaine to the diet can induce the production of GR in hens (21), and GR is considered to be involved in regulating lipid metabolism and TG homeostasis (22). The current study was conducted to investigate whether betaine has preventive or mitigating effects on the early FLS formation and further to reveal the underlying mechanism based on hepatic transcriptomics and serum metabolomics technologies. The study aims to provide a theoretical foundation for the prevention of early FLS and potential therapeutic targets in laying hens.

## 2 Materials and methods

### 2.1 Animals

Hy-Line Brown laying hens were all purchased from Julong Poultry Farm (Wugong, Shanxi, China) and experimental procedures in this research were performed in accordance with the Guidelines for Care and Use of Laboratory Animal and approved by the Animal Ethics Committee of the Northwest A&F University (Permit Number: DK202123).

### 2.2 Animal treatment and sample collection

Sixty Hy-line brown hens aged 21-week-old were used in this study and randomly divided into three groups: Con group (basal diet), FLS group (Dexamethasone injection + basal diet) and betaine group (Dexamethasone injection + basal diet with 8 g/kg betaine). DXM was obtained from Chen Xin Pharmaceutical Co., Ltd. (Shandong, China) and Betaine (pure ≥98%) was obtained from Shanghai Yuanye Bio-Technology Co., Ltd. (Shanghai, China). Each group contained 20 laying hens (one bird/cage). After 1 week pre-feeding, birds in the FLS and betaine groups were subcutaneously injected with dexamethasone (DXM) at a dosage of 4.50 mg/kg body weight for 7 days (at 7:30–8:00) once a day, and hens in the Con group was injected with an equal volume of normal saline. All hens were housed in an environmentally controlled room where the temperature was maintained at about 25 ± 2°C, and the relative humidity was controlled from 60 to 70%. The water was provided freely and about 95 g of feed was fed per day on a restricted feeding regimen. The experimental diet was a corn-soybean-basal diet, formulated according to the guidelines provided by the Chinese Feeding Standard of Chickens (NY/T33-2004). The detailed component of basal diet was shown in Table 1. At the end of the trial, 10 birds were randomly selected and weighted. The blood sample was firstly collected from brachial vein under the wing, and serum was obtained after centrifugation at 3,000 × *g* for 10 min. Thereafter hens were sacrificed by cervical dislocation, and the liver was carefully removed and immediately weighed. Meanwhile, hepatic pathological characters were observed and 1 cm^3^ piece of liver was fixed in paraformaldehyde. The rest of the liver was frozen in liquid nitrogen, and then transferred to −80°C for further analysis.

**Table 1:** Composition and nutrient levels of basal diet.

Composition (air-dry basis) %	Basal diet
Corn	56.69
DDGS	4.00
Soybean meal (43%)	25.77
DL-methionine	0.18
Fat-soybean oil	1.51
CaCO3	9.04
CaHPO4 21/16	1.15
NaCl	0.26
Choline chloride (60%)	0.15
Premix*	1.00
Bentonite	0.25
Total	100.00
Nutrient levels	
Metabolizable energy, kcal/kg (calculated)	2,600
Crude protein (calculated)	16.5
Total phosphorus (calculated/analyzed)	0.53/0.49
Non-phytate phosphorus (calculated)	0.32
Calcium (calculated/analyzed)	3.50/3.52

*The composition of premixes: iron, 60 mg; manganese, 60 mg; copper, 8 mg; zinc, 80 mg; selenium, 0.3 mg; iodine, 0.35 mg; vitamin A, 8,000 IU; vitamin D3, 1,600 IU; vitamin E, 30 mg; menadione, 1.5 mg; vitamin C, 200 mg, thiamine, 4 mg; riboflavin, 13 mg; pantothenic acid, 15 mg; nicotinamide, 20 mg; pyridoxine, 6 mg; biotin, 0.15 mg; folic acid, 1.5 mg; cobalamin, 0.02 mg; additional 8,000 mg betaine was added to the betaine group.

### 2.3 Hepatic morphology

The liver was processed in paraffin and stained with hematoxylin–eosin (H&E) and oil red O after fixed in 4% paraformaldehyde for more than 24 h, which were operated by Wuhan Servicebio technology Co., Ltd. (Wuhan, China).


Liver index=wetliver weight÷body weight×100%

### 2.4 Hepatic TC and TG contents determination

Small pieces of liver tissue were cut and homogenized with PBS in high-throughput homogenizer. The supernatant was collected after centrifuged at 3,000 × *g* for 15 min at 4°C and the protein concentration were further detected by BCA commercial kit (Xi’an AccuRef Scientific Co., Ltd., Xi’an, China). Additionally, hepatic total cholesterol (TC), triglyceride (TG) content was measured by commercial kits (Nanjing Jiancheng Institute of Biological Engineering, Nanjing, China) based on kits instructions. Data conversion and standardization were performed based on the protein concentration.

### 2.5 Serum biochemical indicators

Serum biochemical parameters including aspartate aminotransferase (AST), total protein (TP), total bilirubin (TBIL), total biliary acid (TBA), TC, high density lipoprotein cholesterol (HDL-c), low density lipoprotein cholesterol (LDL-c), and glucose (GLU) were determined using 7,180 Clinical Analyzer (Hitachi, Japan) at Yangling Demonstration Zone Hospital (Yangling, China).

### 2.6 Transcriptomics analysis and RT-PCR

Total RNA was extracted from liver samples with Trizol reagent according to the manufacturer’s instructions (TaKaRa, Dalian, China). RNA-seq libraries were constructed and sequenced by Shanghai Personal Biotechnology Co., Ltd. through llumina NovaSeq. The filtered reads were compared to the reference genome (GRCg7b, GCF_016699485.2) using HISAT2^1^ after checking the data quality. The accession number is PRJNA1130356. Then, differential expression genes (DEGs) were identified according to the parameters of log2 fold change >1 and *p*-value <0.05. All DEGs were annotated in the Kyoto Encyclopedia of Genes and Genomes (KEGG)^2^ and KEGG enrichment analysis was performed using cluster profiler (the criterion for significant enrichment was *p*-value <0.05).

For RT-PCR, the total extracted RNA was reversely transcribed to corresponding cDNA according to Evo M-MLV RT Premix for qPCR (Accurate Biotechnology, Co., LTD., ChangSha, China). Some DEGs expression were selected for further verification. *β*-actin was used as internal reference gene, and the sequence of gene primers are shown in Table 2. The calculation method and reference system were referred to our previous study (23).

**Table 2:** Forward and reverse primer sequences for RT-PCR analysis.

Gene	Accession	Primer sequences, 5′–3′	Product size, bp
*β*-actin	L_08165	F: ATTGTCCACCGCAAATGCTTC R: AAATAAAGCCATGCCAATCTCGTC	113
ACC	XM_046929960	F: GCTTCCCATTTGCCGTCCTA R: GCCATTCTCACCACCTGATTACTG	185
FAS	NM_205155	F: TTTGGTGGTTCGAGGTGGTA R: CAAAGGTTGTATTTCGGGAGC	212
SCD1	NM_204890	F: GTTTCCACAACTACCACCATACATT R: CCATCTCCAGTCCGCATTTT	175
ELOVL6	XM_046916529	F: GGTGGTCGGCACCTAATGAA R: TCTGGTCACACACTGACTGC	169
SREBP1	XM_046927256	F: GCCCTCTGTGCCTTTGTCTTC R: ACTCAGCCATGATGCTTCTTC	130
GR	NM_001037826	F: GGAAACCTGGGGGAAGACTG R: TCACTTGAGGCATCGGCATT	245
ATGL	NM_001113291	F: TCCTAGGGGCCTACCACATC R: CCAGGAACCTCTTTCGTGCT	195
MTTP	NM_001109784	F: GCAGATGGACAGAGTTGGCT R: ACACCAAAAGTGCAAGGTGC	224
APOC3	NM_001302127.2	F: CCGAAGCTCCCGATAAGACAG R: CCGTCTTGACTGCCTCAGTG	81
GPX4	NM_001163232.3	F: AAAGTACGCGGGGAAGATGG R: CCCAAATTGGTTGGAGGGGA	145
APOA4	NM_204938.3	F: GTACTTCACTGAGCTGGGCA R: CTCCTCGGCGTATGAGTTCG	127
GOS2	NM_001190924.4	F: GCCCAACAGGAAGATGGTGA R: ACGACTTCTTGCTCTGCTCC	203
ERG28	XM_040672773.2	F: TCCGCAACCAAACCCTCTAC R: GGTACTGTAGCCCGATCAGC	171
PLA2G3	XM_040684216.2	F: CTCCGAGCTGGGTCTGTTCC R: TAGTTGCGGATGCCGAAGTT	105
SLC5A8	XM_040657436.2	F: TGGTAAGAGTGCTGTCCCCT R: ATGCTCTCGGGATCAGTTCT	197

### 2.7 Metabolomics analysis

Metabolites extraction was following the method previously described (24), and performed with Vanquish UHPLC System (Thermo Fisher Scientific, United States) and Orbitrap Exploris 120 (Thermo Fisher Scientific, United States). Next, based on the previous reports, the original GC–MS data were selected, aligned and a series of processing was performed (25). Orthogonal partial least-square discriminant analysis (OPLS-DA) was performed using Ropls software and used for screening differential metabolites based on *p*-value <0.05, variable importance projection (VIP >1) and fold change (FC > 2). The identified differential metabolites were employed for functional pathway enrichment using MetaboAnalyst and the criterion for significant enrichment was *p*-value <0.05.

### 2.8 Western blot analysis

The frozen liver tissue was first lysed and homogenized in cold RIPA lysis buffer with protease inhibitor and phosphatase inhibitor (DIYIBio, Shanghai, China). BCA kit (Xi’an AccuRef Scientific Co., Ltd., Xi’an, China) was employed to detect the protein concentration in supernatant liquid after centrifugation. SDS-polyacrylamide gel electrophoresis was engaged to protein separation and further transferred to a polyvinylidene difluoride (PVDF) membrane. After that, the blots were blocked in 5% BSA before being incubated with the following primary antibodies: *β*-actin (PTMBIO, PTM-5028, Hangzhou, China), PI3K (Abways, CY6915), AKT (Abways, CY5561, Shanghai, China), P-AKT (Abways, CY6569, Shanghai, China), P53 (Abways, CY5131, Shanghai, China), SREBP1c (Wanleibio, wl01314, Shenyang, China), C/EBPα (Abways, CY5723, Shanghai, China), FTO (Abways, CY7205, Shanghai, China), which were diluted at a ratio of 1:1000. The secondary antibody obtained from DIYIBio (Shanghai, China) was used after 1:2000 dilutions. Images were quantified by Image J (National Institutes of Health, MD).

### 2.9 Statistical analysis

All data are analyzed by unpaired Student’s t test using GraphPad Prism 8 (United States) to compare the statistically significant differences between Con vs. FLS and FLS vs. BT groups and shown as mean ± SEM, and *p*-value <0.05 was considered to be statistically significant.

## 3 Results

### 3.1 Phenotypic observation of hepatic lipid metabolism

**Figure 1:**
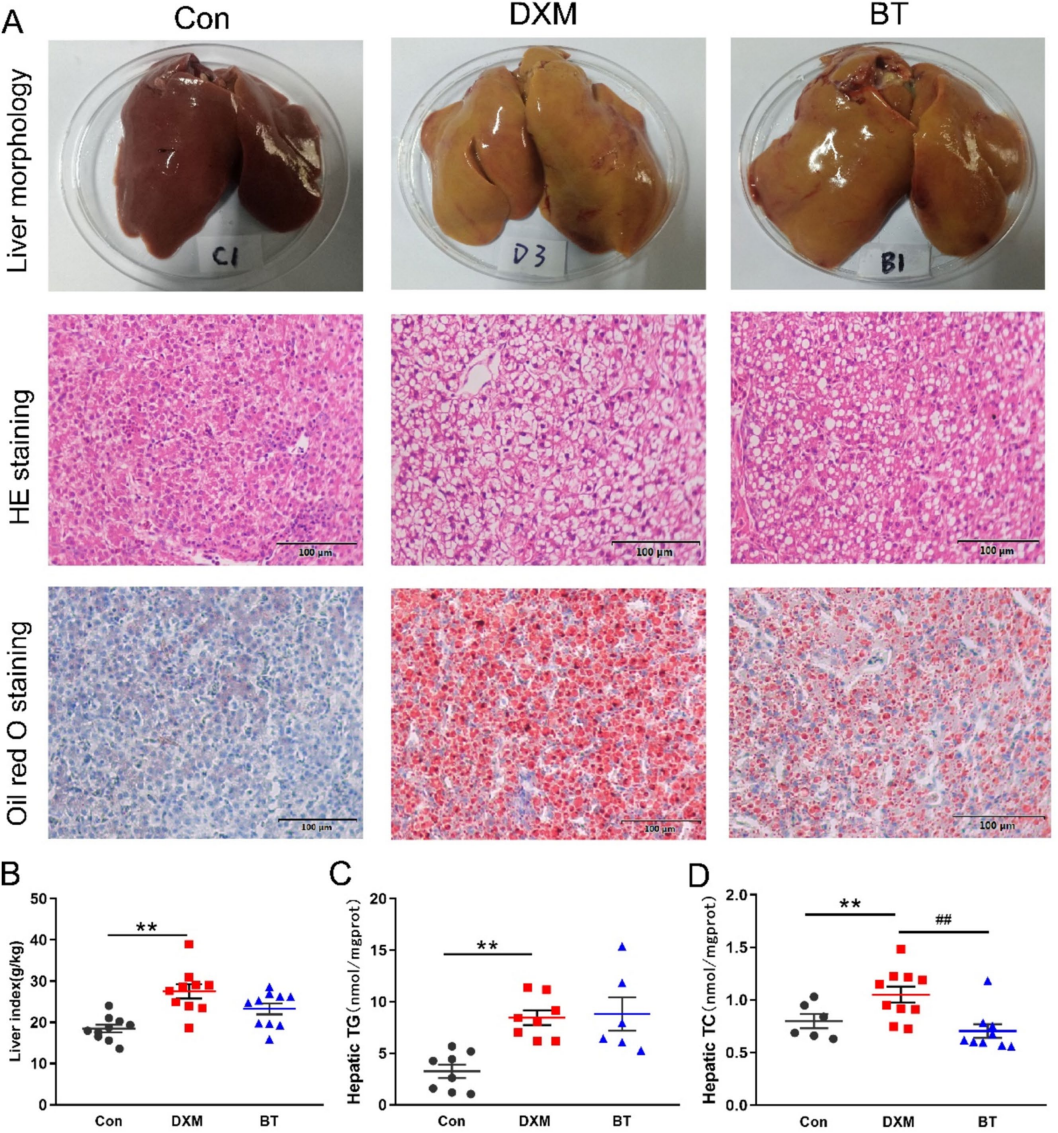
Phenotypic observation of hepatic lipid metabolism. (A) Liver histological sections assessed by morphological observation, H&E and oil Red O staining (200×, 100 μm) in different groups. (B) Liver index. (C) Hepatic TG. (D) Hepatic TC. Data were expressed as mean ± SEM (*n* = 10). *p < 0.05, **p < 0.01 represent that the comparison between Con and FLS group is statistically significant. ^#^*p* < 0.05, ^##^*p* < 0.01 represent there is statistical significance between FLS and BT group.

As presented in Supplementary Figure S1, DXM injection had no effect on body weight and average daily feed intake. The appearance of the liver and pathological examination stained with H&E and oil red O are shown in Figure 1A. The color of liver in Con group was bright red, while it was yellowish and fragile in FLS group. H&E and oil red O staining revealed significant vacuole and lipid droplet accumulation in the liver of FLS group, but dietary betaine addition could reduce this accumulation to some extent. Moreover, when compared to the Con group, the liver index, hepatic TG and TC contents increased dramatically in the FLS group (*p* < 0.05), whereas the TC content was significantly reduced in betaine group (*p* < 0.05; Figures 1B–D).

### 3.2 Serum biochemical characteristics

**Figure 2:**
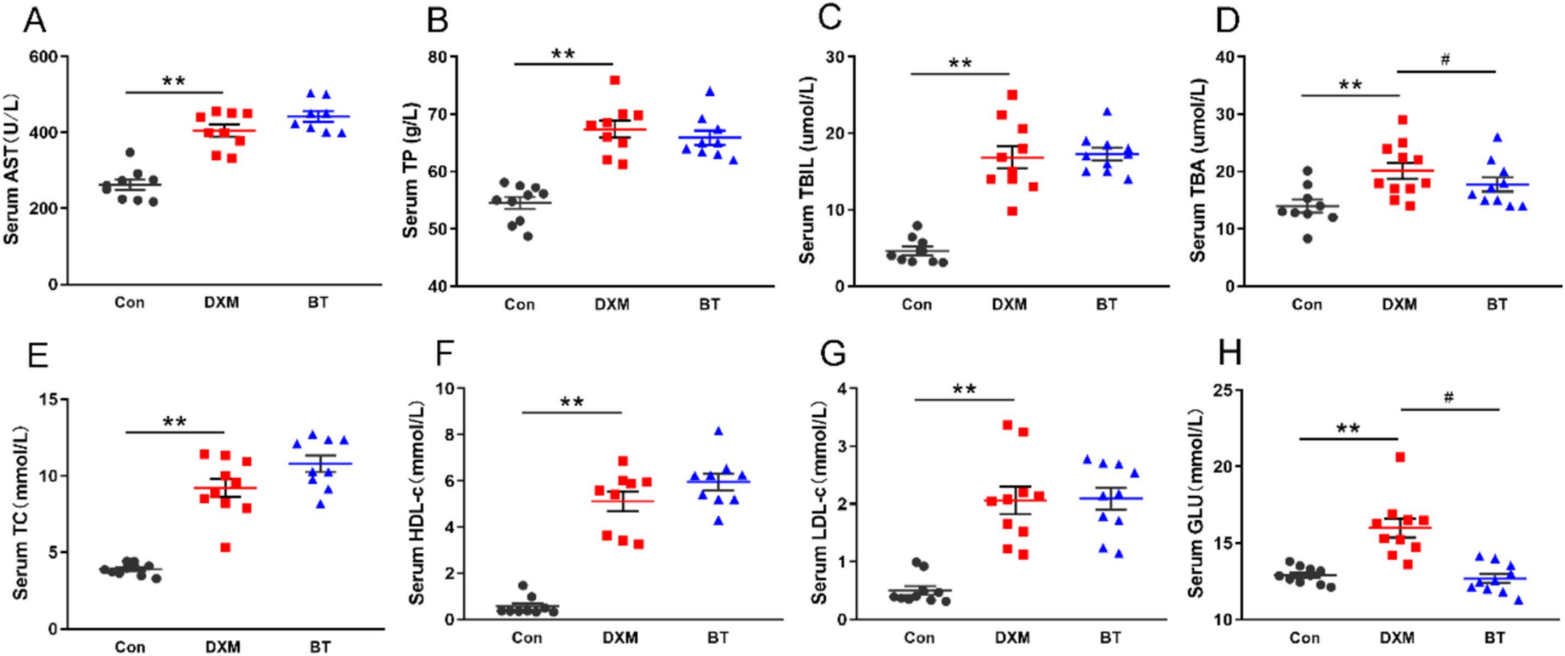
Serum biochemical parameters for AST, TP, TBIL, TBA, TC, HDL-c, LDL-c and GLU. Data were expressed as mean ± SEM (*n* = 10). *p < 0.05, **p < 0.01 represent that the comparison between Con and FLS group is statistically significant. ^#^*p* < 0.05, ^##^*p* < 0.01 represent there is statistical significance between FLS group and BT group.

As shown in Figure 2, DXM administration markedly increased serum AST, TP, TBIL, TBA, TC, HDL-c, LDL-c, GLU concentration (*p* < 0.01) when compared with the control. Conversely, betaine could significantly reduce the TBA and GLU levels compared with FLS group (*p* < 0.05).

### 3.3 DEGs and KEGG pathway analysis by transcriptomics

**Figure 3:**
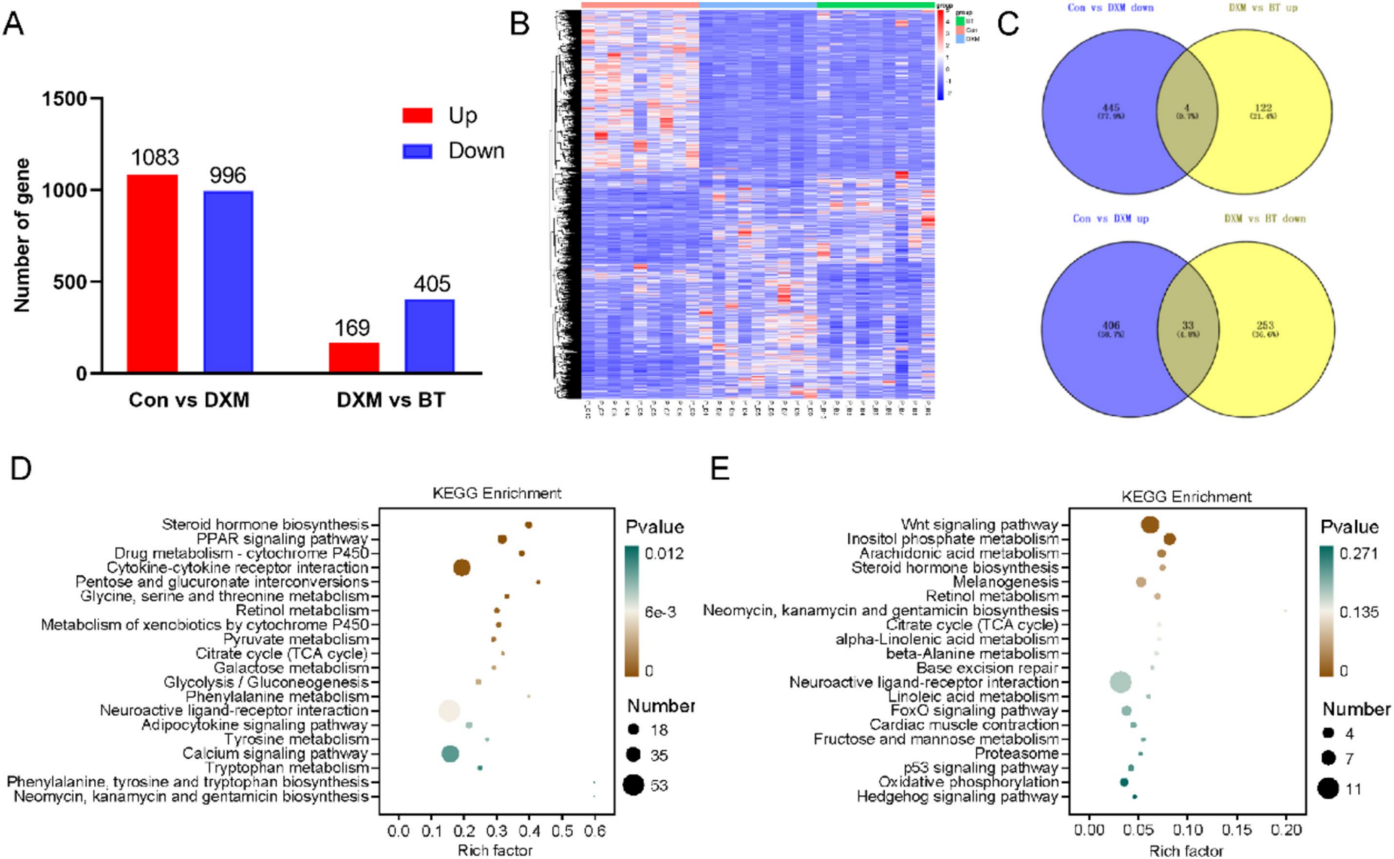
Hepatic DEGs identification and KEGG pathway enrichment analysis by transcriptomics. (A,B) The number and heatmap of hepatic DEGs among Con, FLS and BT groups. (C) Venn diagram of DEGs from different comparison groups. (D,E) Pathway enrichment based on DEGs from Con vs. FLS and FLS vs. BT, respectively.

To further investigate the potential mechanism of betaine in preventing FLS in laying hens, DEGs were identified through RNA-seq. A total of 1,083 and 996 up- and down-regulated genes were identified, respectively, between the Con and FLS group. Meanwhile, 574 DEGs were identified between the FLS and betaine groups, including 169 upregulation and 405 downregulation (Figure 3A). Cluster analysis of DEGs heatmap showed that the addition of betaine could rescue some DEGs disturbed by DXM injection (Figure 3B). Overlapped analysis identified 37 genes rescued by betaine including APOC3, APOA4, G0S2, ERG28, PLA2G3, GPX4, and SLC5A8, which are related to lipid metabolism and antioxidant functions (Figure 3C). Total DEGs between Con and FLS group significantly enriched steroid hormone biosynthesis, PPAR signaling pathway, pyruvate metabolism, citrate cycle (TCA cycle), glycolysis/gluconeogenesis and adipocytokine signaling pathways (Figure 3D). Similarly, pathways such as arachidonic acid metabolism, citrate cycle (TCA cycle), alpha-linolenic acid metabolism, linoleic acid metabolism p53 signaling pathway, FoxO signaling pathway and oxidative phosphorylation pathways were enriched in DEGs between FLS and betaine groups (Figure 3E). To further explore metabolic changes clearly, up- or down-regulated DEGs were employed for enrichment analysis separately (Supplementary Figure S2). Pathways including PPAR signaling pathway, glycolysis/gluconeogenesis, galactose metabolism, pentose and glucuronate interconversions, citrate cycle (TCA cycle), fatty acid biosynthesis and insulin signaling pathway were enriched by up DEGs between Con and FLS groups. Conversely, several metabolic processes were predicted to be changed based on downregulated DEGs between FLS and betaine groups, such as alpha-linolenic acid metabolism, p53 signaling pathway, linoleic acid metabolism, fructose and mannose metabolism, mTOR signaling pathway and oxidative phosphorylation.

### 3.4 Differential metabolites analysis based on serum metabolomics

**Figure 4:**
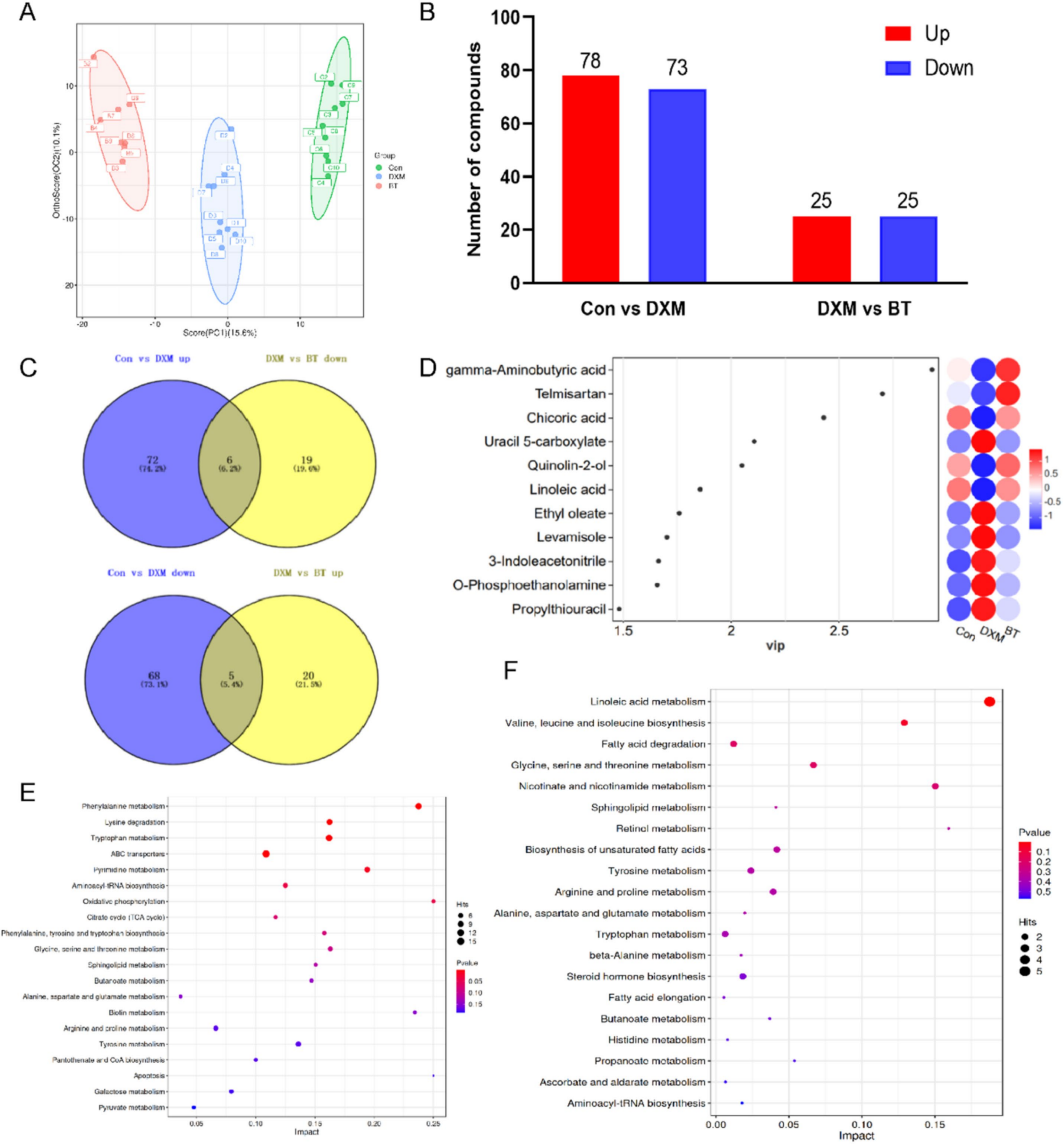
Differential metabolites analysis based on serum metabolomics. (A) OPLS-DA score map of serum metabolites combining positive and negative ions. (B) The number of differential metabolites among Con, FLS and BT groups. (C) Venn diagram of differential metabolites from different comparison groups. (D) VIP value map of differential metabolites rescued by BT. (E) Pathway enrichment based on differential metabolites from Con vs. FLS and FLS vs. BT, respectively.

The OPLS-DA score plot distinctly showed separation among Con, FLS and BT betaine groups (Figure 4A). Compared to the Con group, 78 up- and 73 down-regulated metabolites were identified in FLS group. Similarly, a total of 50 differential metabolites were observed in betaine group in comparison with those in FLS group (Figure 4B; Supplementary Table S1). A total of 11 metabolites were found to be rescued by dietary betaine addition (Figures 4C,D), including 3-Indoleacetonitrile, Ethyl oleate, Levamisole, O-Phosphoethanolamine, Propylthiouracil, Uracil 5-carboxylate, Chicoric acid, gamma-Aminobutyric acid, Linoleic acid, Quinolin-2-ol and Telmisartan. Differential metabolites between Con and FLS groups fall in pathways such as pyruvate metabolism, galactose metabolism, citrate cycle (TCA cycle), oxidative phosphorylation, apoptosis. However, fatty acid elongation, biosynthesis of unsaturated fatty acids, fatty acid degradation, linoleic acid metabolism and ascorbate and aldarate metabolism were enriched by differential metabolites between FLS and betaine groups (Figures 4E,F).

### 3.5 DEGs validation and protein expression

**Figure 5:**
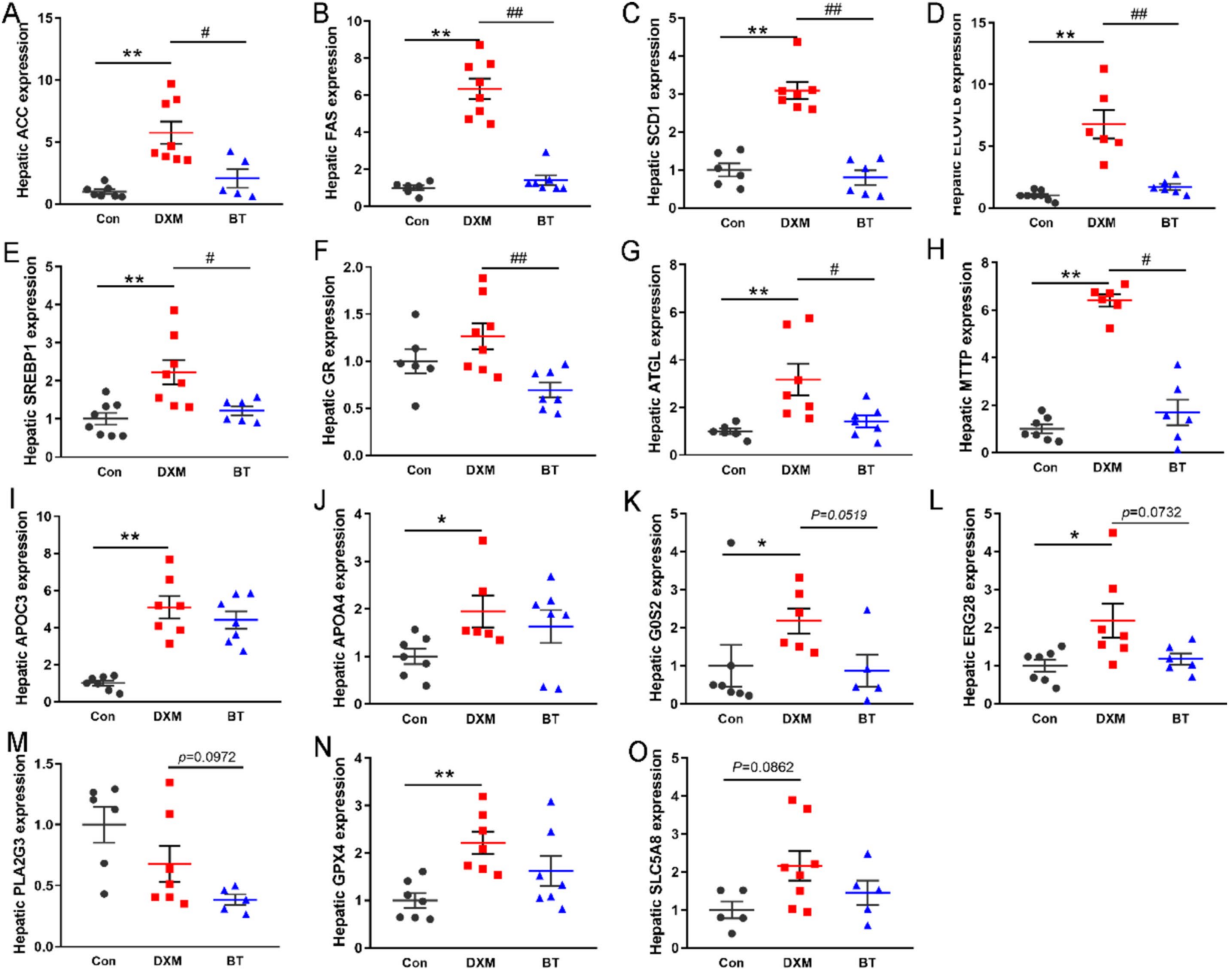
Differential expression genes (DEGs) validation expression. (A–H) The effects of BT on gene expression associated with *De novo* synthesis and decomposition of fatty acids. (I–O) RT-PCR validation of BT regulate genes. Data were expressed as mean ± SEM (*n* = 10). *p < 0.05, **p < 0.01 represent that the comparison between Con and FLS group is statistically significant. ^#^*p* < 0.05, ^##^*p* < 0.01 represent there is statistical significance between FLS group and BT group.

**Figure 6:**
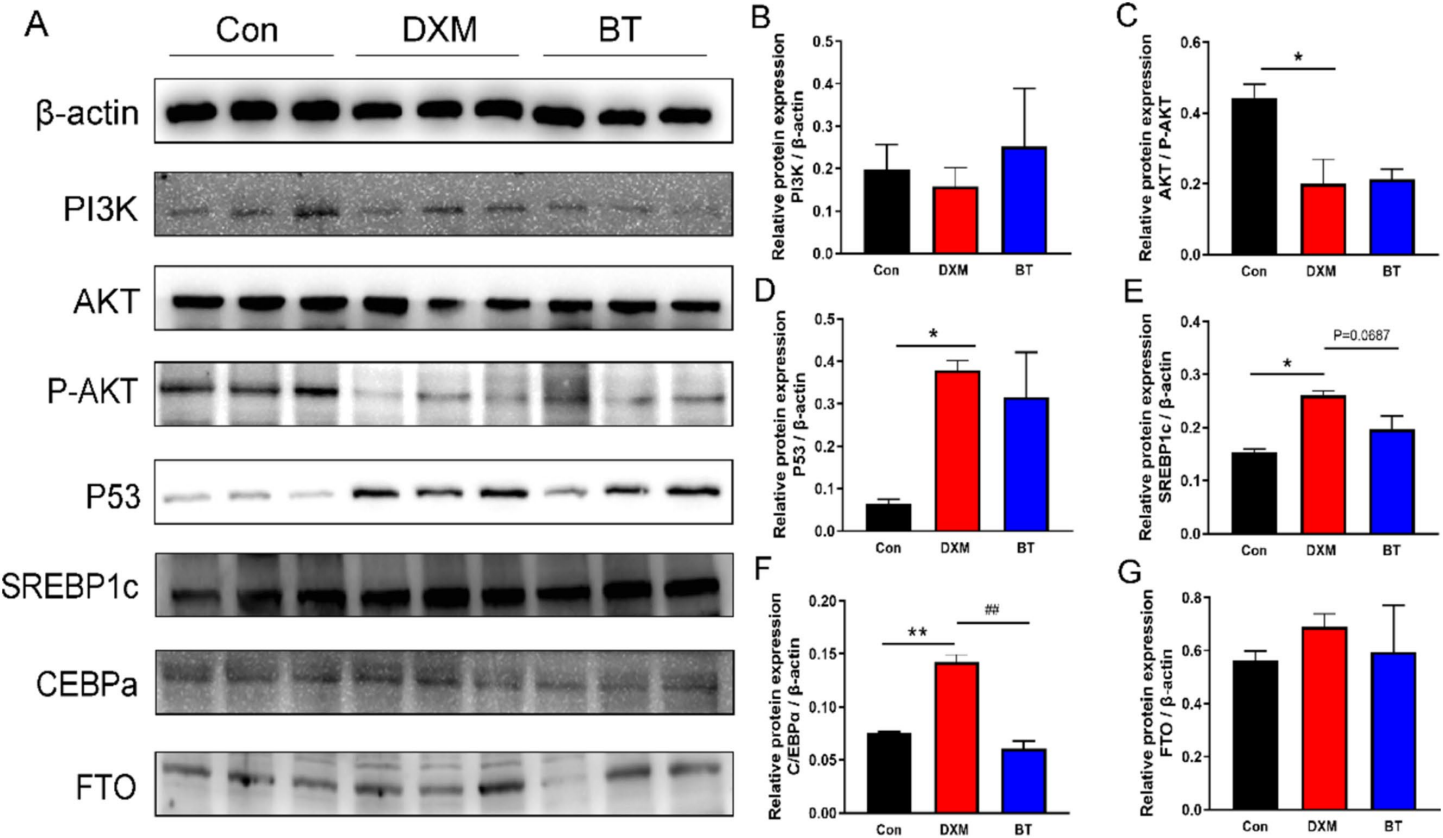
Validation of certain protein expression. (A) Western blot analysis of protein expression in pathways associated with apoptosis and lipogenesis. (B–G) Quantitative analysis of Western blot bands which were normalized to *β*-actin.

To corroborate the findings from the transcriptome analysis, some DEGs were selected for RT-PCR validation. As shown in Figures 5A–H, increased mRNA levels of ACC, FAS, SCD1, ELOVL6, SREBP1, ATGL and MTTP were found in FLS group (*p* < 0.01), while these genes mRNA abundance were reduced by dietary betaine addition (*p* < 0.05). Several genes rescued by betaine such as APOC3, APOA4, G0S2, ERG28, PLA2G3, GPX4 and SLC5A8, exhibited consistency with the findings from the RNA-Seq analysis (Figures 5I–O). In parallel, the abundance of apoptosis related protein p53, and lipogenesis related protein SREBP1c and CEBPα were notably elevated in the FLS group (Figure 6A). However, in comparison to the FLS group, CEBPα protein expression was significantly decreased (*p* < 0.01) and there was a decreasing trend in SREBP1c (*p* = 0.0687) in betaine group (Figures 6B–G).

## 4 Discussion

Fatty liver syndrome, as a common metabolic disease, exhibits a high prevalence during laying periods and its incidence increases with age (3). The hepatic steatosis model induced by DXM had been reported before (26, 27). Excessive DXM injection increases insulin resistance with interference in glucose/insulin homeostasis and escalates liver lipid deposition (28, 29). The heightened lipid deposition in the liver can lead to inflammation, fibrosis and eventual cirrhosis (30). According to previous reports, betaine has been shown to alleviate and prevent FLS by reducing adiponectin levels in the blood and decreasing hepatic oxidative stress (31, 32), as well as inflammation, apoptosis, and metabolic abnormalities (33). In this study, we successfully induced an early FLS model in laying hens without inflammation response. At the same time, during the process of FLS model induction, hepatic lipidosis was notably ameliorated by dietary betaine addition, as evidenced by reduced TC contents and lipid droplets.

When hepatocytes are damaged, AST enters the bloodstream through the hepatocyte membrane, and the bile acid metabolism becomes aberrant, causing elevated TBA and AST levels in serum. In the current study, the AST and TBA levels were significantly increased in FLS group, indicating the occurrence of liver impairment. Additionally, serum higher TP and TBIL levels also implied abnormal liver function. Consistent with previous studies (34–36), we found substantial parameters rise related to lipid metabolism including serum TC, HDL-c, LDL-c and GLU. However, serum higher TBA and GLU levels induced by DXM, was dramatically decreased through dietary betaine treatment. It is hypothesized that betaine may alleviate FLS by modulating bile acid and glucose metabolism.

Transcriptomics analysis provided insights into the molecular mechanisms underlying the preventive effects of betaine on FLS. The results showed that betaine reversed some genes expression such as APOC3, APOA4, G0S2, ERG28, PLA2G3, GPX4, and SLC5A8. APOC3 and APOA4, which belong to lipid transport proteins family and are known to regulate plasma TG levels and inhibit hepatic lipase activity, ultimately affecting VLDL assembly and secretion (37, 38). G0S2 is found to enhance TG accumulation and stimulate the development of fatty liver by binding to ATGL (39). Previous reports have reported the relationship between ERG28 and TC synthesis (40) as well as PLA2G3 (41). Indeed, KEGG pathway analysis revealed enrichment of lipid metabolism-related pathways in the FLS group, such as PPAR signaling pathway, glycolysis/gluconeogenesis, galactose metabolism, pentose and glucuronate interconversions, citrate cycle (TCA cycle), fatty acid biosynthesis, insulin signaling pathway. These findings aligned with the phenotypic characteristics associated with the occurrence of FLS, implying that the FLS model used in this study can simulate FLS laying hens under natural conditions to some extent. Interestingly, no difference was observed in pro-inflammatory cytokines, which may be attributed to the anti-inflammatory function of DXM and the relatively short period of FLS induction in the study. Conversely, the p53 signaling pathway, alpha-linolenic acid metabolism, linoleic acid metabolism, fructose and mannose metabolism, mTOR signaling pathway and oxidative phosphorylation were also enriched from down-regulation DEGs in betaine group. The p53 signaling pathway and mTOR signaling pathway is related to apoptosis and insulin response, respectively (42, 43). The liver is a vital organ where numerous oxidative processes take place and oxidative stress can result in cellular dysfunction, injury, and ultimately cell death. Alpha-linolenic acid and linoleic acid metabolism can cause lipid oxidative damage (44). Conversely, the downregulation of these pathways indicated dietary betaine addition might reduce lipid deposition and oxidative stress in the liver. These findings suggest that betaine may have a protective effect on FLS via regulating lipid deposition and oxidative stress.

Metabolomics analysis further supported the beneficial effects of betaine on FLS prevention. Studies have proved that chicoric acid can mitigated hyperglycemia and dyslipidemia, reversing oxidative stress and inflammation in the liver induced by high-fat-diet (45). Gamma-Aminobutyric acid was reported to acts as a protective agent to against toxin-induced hepatic damages (46). Linoleic acid (47) and telmisartan (48) can significantly improve insulin secretion and reduce lipid accumulation by inhibiting oxidative stress. Additionally, telmisartan exhibited a protective effect against apoptosis induced by high-fat and high-sugar diet (48). An earlier study indicated that FLS is associated with oxidative stress alterations, which in turn causes insulin resistance and free fatty acids production, finally leading to an imbalance in the antioxidative system (49). This imbalance triggered lipid peroxidation, impaired VLDL secretion and resulted in hepatic TG accumulation (50). In the current study, these metabolites mentioned above were reduced in the FLS group, indicating that the liver is under oxidative stress. A favorable increase in these metabolites was observed in betaine group, suggesting the protective effect of betaine on hyperglycemia, dyslipidemia and oxidative stress. Furthermore, the enriched pathways from differential metabolites between FLS and betaine groups were linoleic acid metabolism, fatty acid degradation, ascorbate and aldarate metabolism. Previous studies have suggested that linoleic acid possesses significant antioxidant effects (51) and ascorbate is recognized as a crucial antioxidant function (5). These findings implied that the therapeutic effect of betaine on FLS might be attributed to its antioxidant regulation.

**Figure 7:**
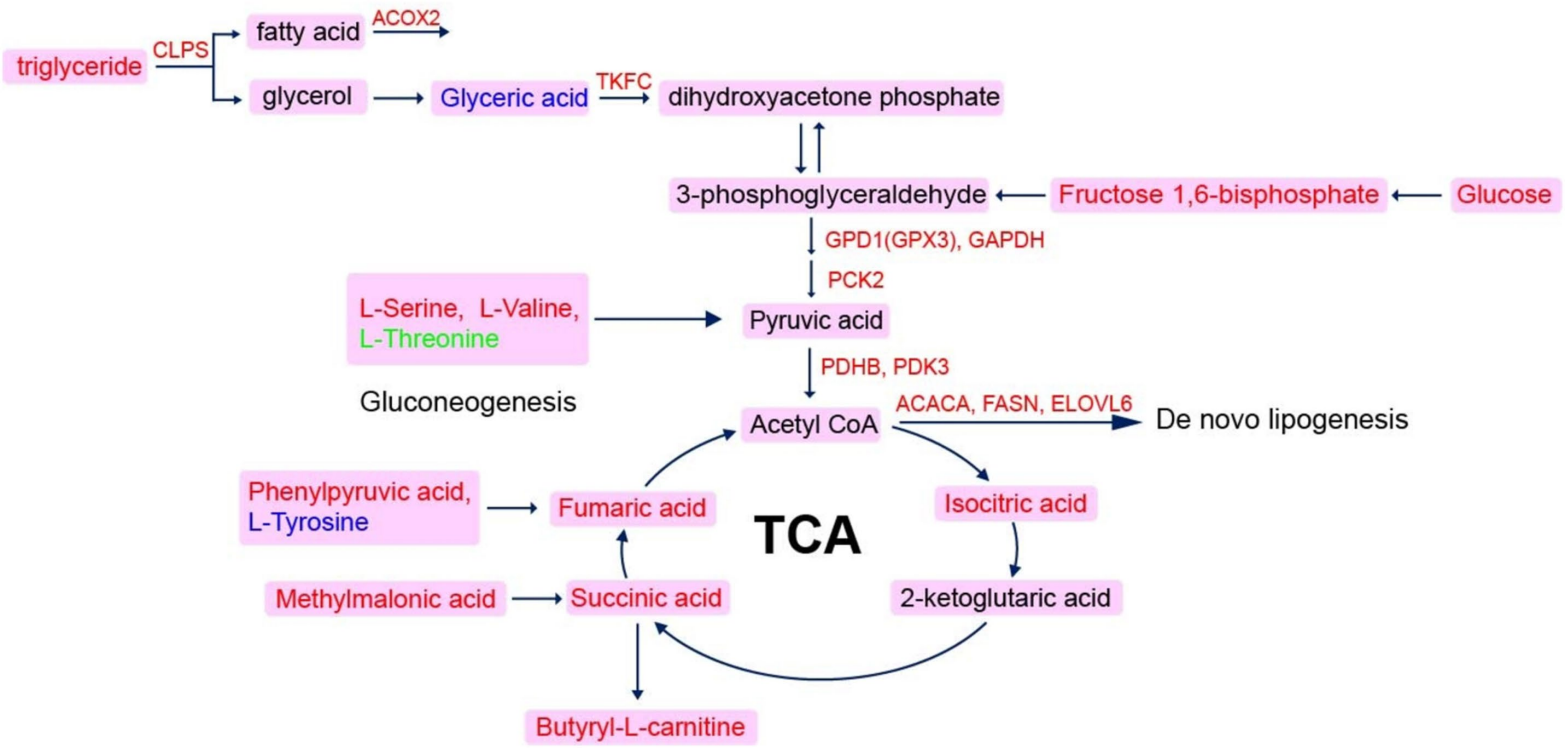
Network diagram of co-regulation with genes and metabolites. The scarlet letters represent genes in liver are up-regulated in FLS group, and pink frame the up-regulation of serum metabolites in FLS group.

It has been reported that an elevation of fatty acid delivery causes an increase in TCA cycle flux, leading to heightened hepatic oxidative stress and inflammation (52). The TCA cycle is a pivotal component of energy metabolism *in vivo* and represents a hub metabolic pathway of carbohydrates, fats and proteins. Consequently, we performed a joint analysis of transcriptomics and metabolomics based on the center of the TCA cycle (Figure 7). The increase abundance of TC, isocitric acid, succinic acid and fumaric acid indicated an elevation in the flow of the TCA cycle, suggesting that hepatic lipid metabolism was disturbed. An intermediate of the TCA cycle, the increase of fumaric acid in the FLS group implied impaired mitochondrial function (5). Meanwhile, elevated serine and valine levels also suggested enhanced gluconeogenesis. Furthermore, genes upregulation related to *de novo* lipid synthesis including ACACA, FASN, and ELOVL6, further supported an increase in fatty acids synthesis. Collectively, TCA cycle metabolism might be a marker of the early stage of FLS, and lipid deposition promoted the oxidative stress, which finally leaded to liver injury. Betaine could develop protective effects on the early fatty liver through ameliorating lipid metabolism and oxidative stress.

## 5 Conclusion

In summary, DXM can induce hepatic lipid accumulation by changing gene expression related to lipid metabolism, and established early FLS model with liver damage. The preventive effects of betaine on FLS mainly attribute to its regulation function of lipid metabolism and antioxidative roles. These findings offer valuable insights into the potential therapeutic application of betaine in preventing FLS in laying hens.

## Data Availability

The original contributions presented in the study are included in the article/Supplementary material, further inquiries can be directed to the corresponding author.

## Footnotes

^1^ http://ccb.jhu.edu/software/hisat2/index.shtml

^2^ http://www.kegg.jp/
