# Supplementary material for: Protective effects of betaine on the early fatty liver in laying hens through ameliorating lipid metabolism and oxidative stress
Source: Front Nutr. 2024 Nov 25;11:1505357. doi: 10.3389/fnut.2024.1505357 (PMC11627039; doi:10.3389/fnut.2024.1505357)
Supplement: Supplementary file 1 [file Image_1.pdf]

Supplemental figures

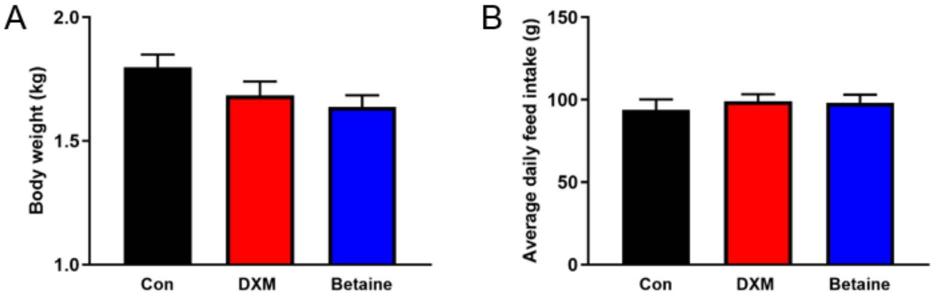

Figure S1 Body weight and average daily feed intake

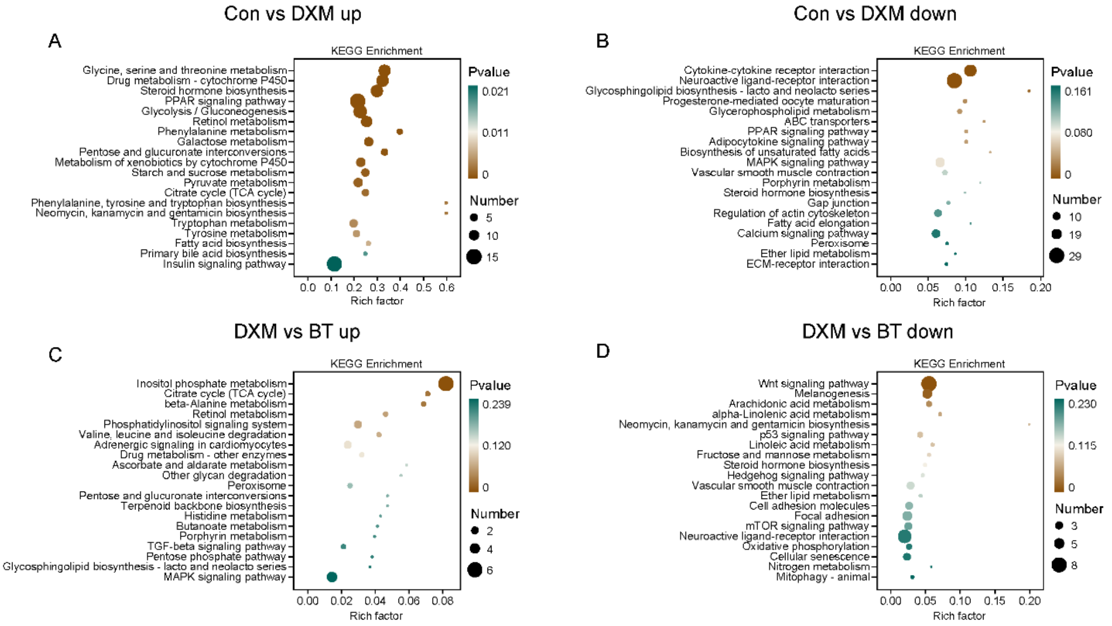

Figure S2 KEGG analysis of up and downregulated DEGs. (A) KEGG pathways enriched form up-regulation genes in Con and DXM groups. (B) KEGG pathways enriched form down-regulation genes in Con and DXM groups. (C) KEGG pathways enriched form up-regulation genes in DXM and BT groups. (D) KEGG pathways enriched form down-regulation genes in DXM and BT groups.
