# Supplementary material for: Protective effects of betaine on the early fatty liver in laying hens through ameliorating lipid metabolism and oxidative stress
Source: Front Nutr. 2024 Nov 25;11:1505357. doi: 10.3389/fnut.2024.1505357 (PMC11627039; doi:10.3389/fnut.2024.1505357)
Supplement: Supplementary file 2 [file Table_1.docx]

Supplementary Table 1 Differential metabolites between the control and betaine addition groups

| Metabolites | VIP^1^ | *P*-value^2^ | Trend |
| --- | --- | --- | --- |
| up | | | |
| 2,3-Butanediol | 2.61 | 0.00 | ↑ |
| 2-Methylene-4-oxopentanedioic acid | 1.88 | 0.03 | ↑ |
| 3-Ketosphingosine | 1.99 | 0.02 | ↑ |
| 4-Hydroxycinnamoylagmatine | 1.98 | 0.02 | ↑ |
| 5-Oxoavermectin ''1b'' aglycone | 2.01 | 0.01 | ↑ |
| Adrenosterone | 1.97 | 0.04 | ↑ |
| Butyryl-L-carnitine | 2.63 | 0.00 | ↑ |
| Cerulenin | 1.76 | 0.04 | ↑ |
| Chicoric acid | 2.50 | 0.00 | ↑ |
| Corticosterone | 2.08 | 0.03 | ↑ |
| Ergothioneine | 1.69 | 0.05 | ↑ |
| Galactaric acid | 2.26 | 0.01 | ↑ |
| gamma-Aminobutyric acid | 2.64 | 0.00 | ↑ |
| Linoleic acid | 1.70 | 0.03 | ↑ |
| L-N2-(2-Carboxyethyl)arginine | 2.04 | 0.01 | ↑ |
| L-Olivosyl-oleandolide | 1.79 | 0.03 | ↑ |
| Miglitol | 1.97 | 0.02 | ↑ |
| N2'-Acetylgentamicin C1a | 1.99 | 0.01 | ↑ |
| Nicotinic acid | 1.63 | 0.05 | ↑ |
| p-Hydroxyphenylacetic acid | 1.90 | 0.03 | ↑ |
| Quinolin-2-ol | 2.10 | 0.01 | ↑ |
| Retinol | 1.71 | 0.03 | ↑ |
| Telmisartan | 2.49 | 0.00 | ↑ |
| Ursodeoxycholic acid | 1.75 | 0.03 | ↑ |
| Vanillin | 2.01 | 0.02 | ↑ |
| down | | | |
| (+)-cis-Isopulegone | 2.23 | 0.00 | ↓ |
| 10E,12Z-Octadecadienoic acid | 1.65 | 0.05 | ↓ |
| 12,13-DHOME | 2.48 | 0.00 | ↓ |
| 13-L-Hydroperoxylinoleic acid | 2.08 | 0.02 | ↓ |
| 1-Hexadecanol | 2.15 | 0.01 | ↓ |
| 2-Aminophenol | 1.83 | 0.03 | ↓ |
| 2-Ketobutyric acid | 1.79 | 0.03 | ↓ |
| 3-Indoleacetonitrile | 2.06 | 0.01 | ↓ |
| 4-Hydroxyphenylacetaldehyde | 1.74 | 0.03 | ↓ |
| 5-Methylcytosine | 2.10 | 0.01 | ↓ |
| 9,10-Epoxyoctadecenoic acid | 2.48 | 0.00 | ↓ |
| Acetylcysteine | 1.74 | 0.03 | ↓ |
| Daidzein | 1.71 | 0.04 | ↓ |
| Ethyl oleate | 1.69 | 0.05 | ↓ |
| Hydroxykynurenine | 1.67 | 0.04 | ↓ |
| Kyotorphin | 1.87 | 0.03 | ↓ |
| Levamisole | 1.85 | 0.03 | ↓ |
| L-Threonine | 2.93 | 0.00 | ↓ |
| m-chlorophenylpiperazine (m-CPP) | 2.10 | 0.01 | ↓ |
| Methyleugenol | 2.02 | 0.01 | ↓ |
| O-Phosphoethanolamine | 2.16 | 0.02 | ↓ |
| Palmitic acid | 1.99 | 0.02 | ↓ |
| Propylthiouracil | 2.15 | 0.01 | ↓ |
| trans-Ferulic acid | 3.15 | 0.00 | ↓ |
| Uracil 5-carboxylate | 2.04 | 0.01 | ↓ |

^1^VIP, Variable Importance in the Projection, was obtained from the OPLS-DA model.

^2^ The *P* value was calculated from Student’s t test.
